# Controlled dynamic stability as the next step in “biologic plate osteosynthesis” - a pilot prospective observational cohort study in 34 patients with distal tibia fractures

**DOI:** 10.1186/1754-9493-8-3

**Published:** 2014-01-21

**Authors:** Thomas Freude, Steffen Schröter, Christoph Emanuel Gonser, Ulrich Stöckle, Yves P Acklin, Dankwart Höntzsch, Stefan Döbele

**Affiliations:** 1 Department of Traumatology and Reconstructive Surgery, BG Trauma Center, University of Tübingen, SS 95, D-72076 Tübingen, Germany; 2 Department of Traumatology, Kantonsspital Graubünden, SS 170, CH-7000 Chur, Schweiz

**Keywords:** Dynamic locking screw, Locking compression plate, Controlled dynamic stability, Bridging plate technique biological internal fixation, Biological internal fixation

## Abstract

**Introduction:**

Delayed bone healing is an eminent problem in the operative treatment of distal tibia fractures. To address this problem from a biomechanical perspective, the DLS 3.7 (Dynamic Locking Screw 3.7 mm) as a new generation of locking screws has been developed. This screw enables the surgeon to control the rigidity of the plate osteosynthesis and thereby to expand clinical options in cases where the bridge plating is chosen for fracture treatment.

**Purpose:**

The purpose of the present prospective study was to evaluate the safety use of the DLS 3.7 in distal tibia fractures where bridge plating osteosynthesis is recommended.

**Methods:**

In a prospective non-controlled cohort study, 34 patients with acute distal tibia fractures (AO 43 A-C) were treated with an angular stable plate fixation using DLS 3.7 or LHS 3.5. Follow-up examinations were performed three, six, twelve, and twenty-four weeks postoperatively and all registered complications were carefully collected.

**Results:**

A total of 34 patients were prospectively enrolled in this study with a minimum follow-up of 6 months or obvious osseous consolidation at an earlier stage. No complications directly related to the DLS 3.7 were recorded and no infections were observed.

**Conclusions:**

This observational study could show that the DLS 3.7 in combination with locking compression plates provides a secure and easy application. According to the recent literature inter-fragmentary micro-motion is one evident goal to increase the reliability in fracture healing. The new DLS 3.7 with a maximum micro-motion of 0.2 mm combines the advantage of micro-motion with the well-known advantages of angular stable plate fixation.

## Introduction

Over the last decades, the state-of-the-art in plate osteosynthesis has evolved in parallel with the general understanding of bone healing. Fifteen years ago, Otmar Trentz postulated that “a fracture is defined as a soft tissue injury, in which we find an associated broken bone just by chance”. Against this backdrop, the technique of open and accurate reduction was gradually replaced by the minimally invasive bridging plate osteosynthesis. In 2003, Perren [1,2] described the bridging plate technique by using angular stable implants. The aim of this technique consisted in preserving the local regeneration potency by protecting the soft tissue in order to reduce the high rate of local complications. This was a logical step towards a biological plate osteosynthesis. Absolute stability was replaced by relative stability and direct bone healing was replaced by indirect bone healing.

Goodship, Kenwright and other authors have analyzed the influence of micromovement upon the healing of tibial fractures. They has been able to demonstrate that the reduction of the axial stiffness leads to a significant increase of the rate of fracture healing [3-12]. Potential changes in the construct, such as a longer plate, different screw configurations, or the variation of the number of cortices of fixation per screw have been proposed to alter the mechanical behavior of fracture fixation constructs and to affect micro-motion and callus formation [4,5,13,14].

**Figure 1:**
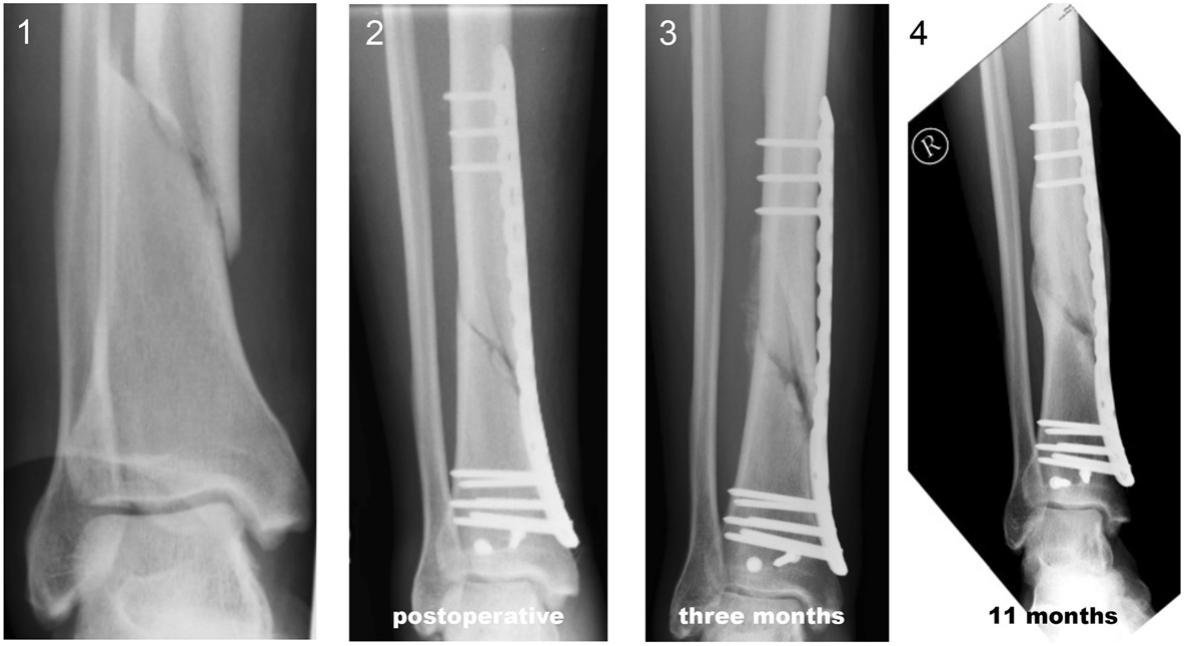
**Distal tibia fracture of a 38-year-old man treated with a distal tibia LCP and conventional locking screws.** Distal tibia fracture: postoperative (1), six-weeks follow-up (2), three-months follow-up (3), and eleven-months follow-up (4), with incomplete consolidation.

Especially simple fractures, i.e. oblique fractures of the distal tibia (AO 42 A2/A3 and AO 43 A1) present an unequal distribution of callus formation. In 2009, the clinical and biomechanical studies about delayed bone healing in distal femur fractures that had been carried out by Bottlang [1], proved that a continuous micro-movement in the fracture zone is necessary to enable sufficient callus formation. Figure 1 presents an example of a unsuccessful treatment of a distal tibia fracture with a low bend medial distal tibia LCP 3.5 (DePuy-Synthes, Zuchwil, Switzerland).

**Figure 2:**
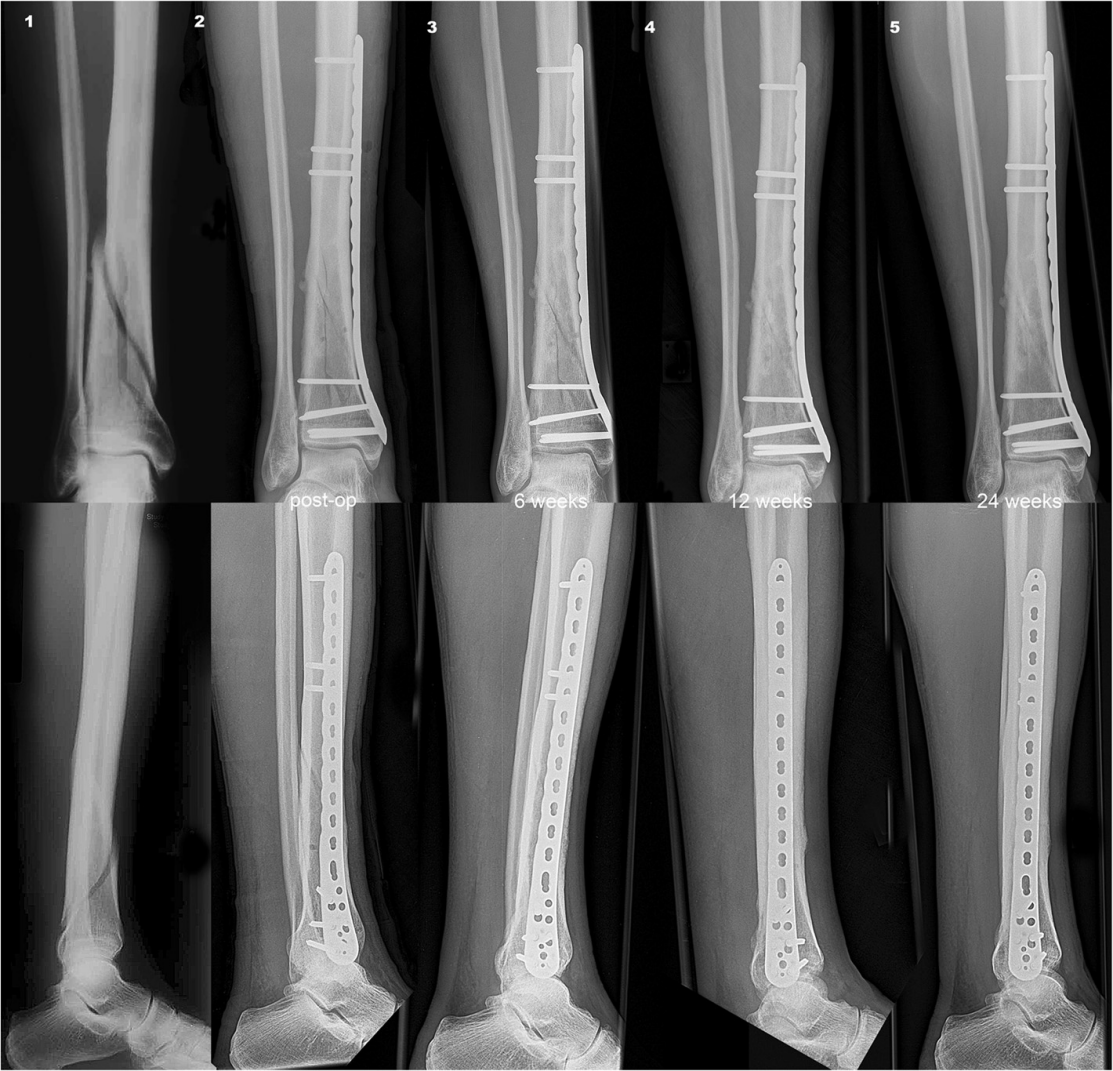
**Distal tibia fracture of a 67-year-old man treated with a distal tibia LCP and DLS 3.7 in the proximal fragment.** Distal tibia fracture: postoperative (1), six-weeks follow-up (2), three-month follow-up (3), and six months follow-up (4) with complete consolidation.

**Figure 3:**
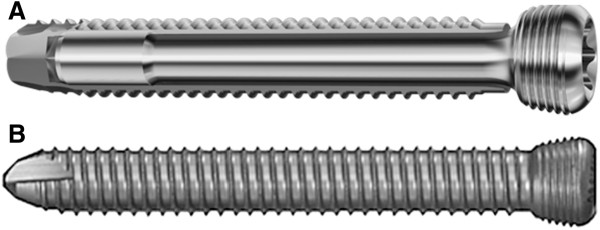
**A. PIN Sleeve Design of the Dynamic Locking Screw (DLS 3.7). B** Locking Head Screw (LHS 3.5). Images kindly provided by DePuy-Synthes, Zuchwil, Switzerland.

**Figure 4:**
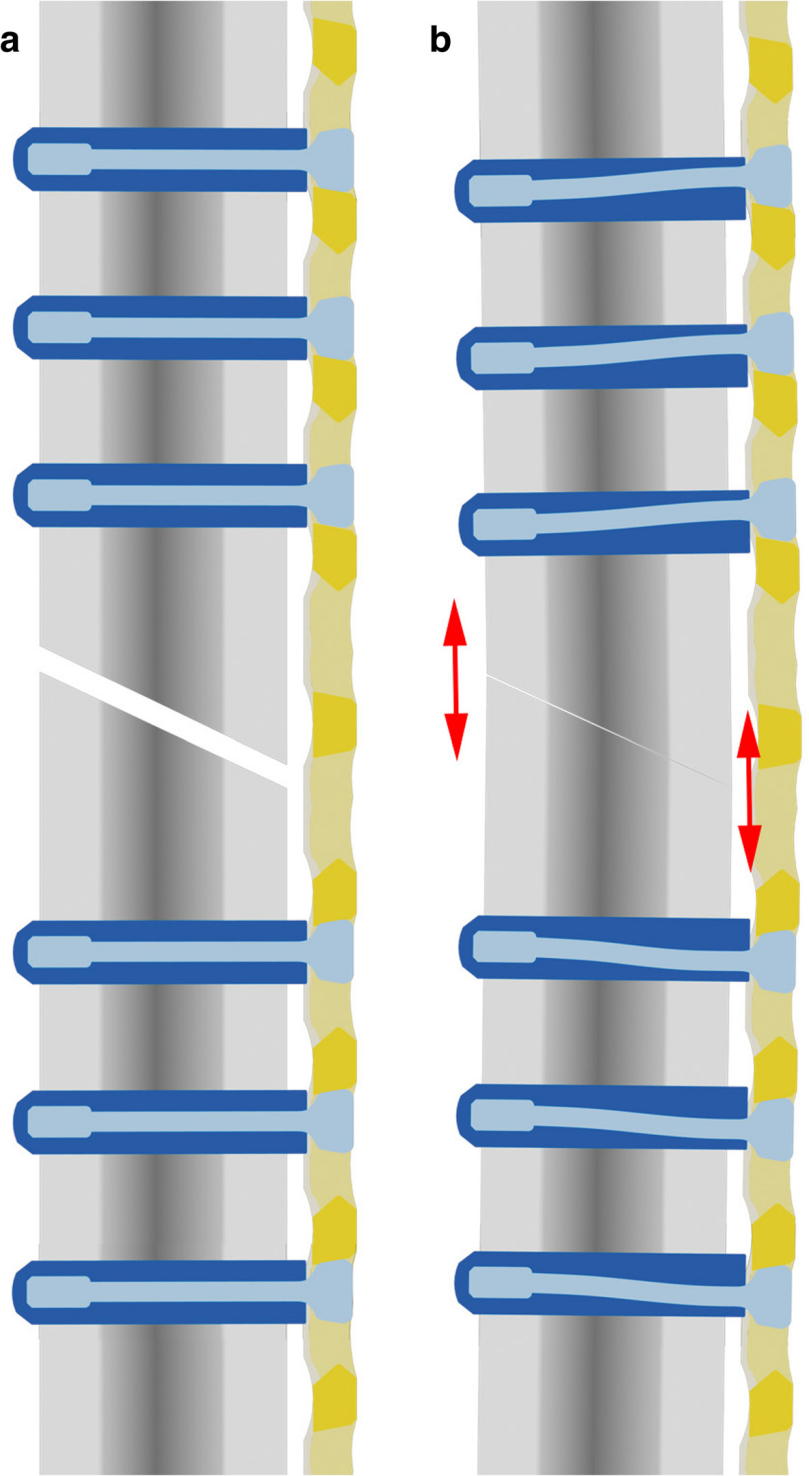
**Schematic graph explaining the underlying biomechanical rationale behind the application of controlled dynamic stability.** Osteosynthesis with a Locking Compression Plate (LCP) in conjunction with the DLS 3.7 **a:** without load and **b:***with* load.

Recently, the Dynamic Locking Screw (DLS 3.7) has been developed by DePuy-Synthes, Zuchwil, Switzerland, to decrease the high rigidity of standard locking plate osteosynthesis Figures 2, 3 and 4.

The purpose of the present study was to show the safe use of a new implant (DLS 3.7) for the treatment of distal tibia fractures in a prospective non-controlled cohort study. The data were collected as part of a multi-center study. In this manuscript, we exclusively present the results obtained from the patients of our own center.

## Materials and methods

Between 03/2009 and 10/2010, a multi-centre study on the plating of acute traumatic distal tibia fracture with low bend distal medial tibia LCP and the DLS 3.7 was conducted. The purpose of this prospective, non-controlled cohort study consisted in evaluating the safe use of the new DLS 3.7. The patients were recruited in four centers (Munich/Germany, Berlin/Germany, Chur/Switzerland and Graz/Austria). Inclusion criteria were acute single fractures of the distal tibia according to the AO Classification (AO 43 A-C), age over 21 years at the time of the trauma, and full compliance to after-care and weight bearing restriction. Exclusion criteria were re-fracture, chronic inflammatory diseases, pregnancy, Hb serum level of less than 10 g/dl at the time of admission, cancer, admission of immune response-modulating drugs, HIV or chronic hepatitis B/C, osteitis or osteomyelitis, head injury, neurological deficit, allergic reaction to the Cobalt-Chromium-Molybdenum alloy (CoCrMo). Moreover, patients with an open fracture with a soft tissue injury worse than grade II according to the Gustilo and Anderson [15] classification were also excluded from our study. All participating patients signed an informed consent. Clinical and radiological follow-up exams were performed after 3, 6, 12, and 24 weeks. The fracture of each patient was classified according to the AO Classification of Fractures. The length of the plate, the type and the number of screws, as well the insertion performance of the DLS 3.7, which was rated as “excellent”, “good”, “satisfactory” or “poor” by the surgeon were documented.

For the evaluation of the safe use of the new implant, implant-specific complications i.e. screw loosening, screw breakage, and screw perforation, and general complications, i.e. pain perception according to the VAS (Visual-Analogue-Scale), were recorded.

For the evaluation of the status of fracture healing, the two-plane radiographs of each patient after 3, 6, 12 and 24 weeks were analyzed by two trauma surgeons who had not participated in the earlier stages of this study. The following parameters were assessed: callus formation at cis-cortex and trans-cortex (“minus”, “plus”, “plus/plus”) and status of consolidation of the fracture (“yes”, “no”). The pictures were blinded for the used implants or screws.

### Dynamic locking screw (DLS 3.7)

The DLS 3.7 is a new type of locking screw that enables the surgeon to control the rigidity of the plating osteosynthesis Figure 3. It builds upon the proven advantages of standard locking screws and eliminates the tension on the bone and the compression between the plate and the bone, while simultaneously retaining the blood circulation and protecting the periosteum from potential damage. The DLS 3.7 consists of a pin with a threaded screw head which is connected to a threaded sleeve based on the angular-stable technique of Locking Screws (Figure 3). The DLS 3.7 is made of a Cobalt-Chromium-Molybdenum alloy (CoCrMo). The material of the screw has been changed in comparison to the LHS 3.5 in order to increase the strength under cyclic forces and to reduce the bone-screw interface for better implant removal. This screw material can be combined with TAN (titanium alloy) or stainless steel plates without any biocompatibility problems.

The design of the screw has been changed considerably While the pin-sleeve part has been left unchanged, the DLS contains a stardrive head and a new blunt screw tip design with five specifically designed drill flutes to improve the cutting performance in combination with a blunt tip to reduce harm to the soft tissue on the trans-cortex.

The increased core diameter of the DLS 3.7 fits all 3.5 LC-Plates. The handling of the drill sleeve and its drillbit has remained unchanged in comparison to the standard instruments used in the placement of the LHS 3.5. The DLS 3.7 can be either inserted with a power tool or manually, but always in combination with a maximum torque of 1.5 Nm. When problems with the insertion of the DLS 3.7 arise, the use of a procedure that has already been established for the LHS 3.5, is recommended.

Biomechanical tests have been able to demonstrate clearly that the DLS 3.7 is able to reduce the rigidity of the screw plate interface [16]. This is a particularly beneficial feature when the bridging plate osteosynthesis is chosen for the fracture treatment.

### Surgical procedure

Surgery was performed within 4 days after the trauma. Either a 3.5 LCP Low Bend Medial Distal Tibia Plate (DePuy-Synthes, Zuchwil, Switzerland) or an LCP Distal Tibia Plate (DePuy-Synthes, Zuchwil, Switzerland) was used in all cases. These plates are intended for the fixation of simple and complex intra- and extra-articular fractures of the distal tibia. The study design did not limit the number of the used LHS 3.5 or DLS 3.7 screws. However, the DLS 3.7 had to be used at least on one side of the fracture, or in the metaphysial and in the diaphysial part of the tibia shaft. A simultaneous use of both types of screws (DLS 3.7 and LHS 3.5) on the same side was not allowed.

In most cases, the minimally invasive approach was used: A short (40–50 mm) incision was made at the medial malleolus and the plate was slid in along the bone. Next, the reduction of the fracture and the positioning of the plate were carried out under fluoroscopy control. On the proximal side of the fracture in the tibia shaft, bi-cortical screw insertion was performed. In the distal juxta-articular part, all screws were fixed monocortically.

#### Implant removal

After complete consolidation of the fracture, the removal of the implants was intended.

## Results

In total, 34 fractures in 34 patients were enrolled in our own center in the contest of the limited multi-center study. The follow-up was 6 months or obvious osseous consolidation at an earlier stage. 5 patients dropped out during the follow-up. In order to classify the fracture type, the AO Classification of Fractures was used. Three different types of fracture were included: AO 43 A (41%), AO 43 B (47%), and AO 43C (12%). The mean age of the patients was 51 years (SD ± 15 years) (44% male and 56% female). In 22 cases, the DLS 3.7 was used in the diaphyseal area only, whereas in 12 cases, DLS 3.7 was used in the proximal as well as in the distal area.

In the proximal part, this resulted in the use of three DLS 3.7 (range: 0 to 4) on average per patient. In the distal part, the mean number of DLS 3.7 per patient was 0.8 (range: 0 to 6). The mean number of LHS 3.5 per patient was 5 (range: 0 to 7). Finally, the mean number of cortical screws was 0.1 (range: 0 to 3) per patient. The distribution of the plate length was 239 mm in 24% of the patients, 213 mm in 26% of the patients, 208 mm in 3% of the patients, 198 mm in 12% of the patients, 187 mm in in 26% of the patients, and 171 mm in 9% of the patients.

The bone quality, which was assessed by the surgeon (1–5, 1 meaning “very good” and 5 meaning “poor”), was classified as follows: 1: 35% of the subjects, 2: 44% of the subjects, 3: 6% of the subjects, 4: 12% of the subjects, and 5: 3% of the subjects. The insertion of the DLS 3.7 was excellent in 50% of the patients, good in 32% of the patients, satisfactory in 15% of the patients, and poor in 3% of the patients. Six weeks after surgery, the mean VAS was 1.4 (SD ± 0.9). At the time of the 3 months follow-up, it had slightly deteriorated to 1.6 (SD ± 1.1). At the time of the 6 months follow-up, it had improved again to 1.5 (SD ± 1.3). After 6 weeks, however, the differences between the three follow-ups were not significant. The radiological assessment by the surgeon was union in 27% and non-union in 73% of the cases. After three months, 78% of the fractures were assessed as union and after 6 months, even 100% of the fractures were assessed as union. The assessment by the observers (authors) that was based on callus formation, led to the following results: After 6 weeks, callus formation was observed in 35% of the fractures. After 3 months, it was even visible in 96% of the fractures. However, after 6 months, callus formation was only visible in 83% of the fractures. This difference can be explained with the dropout of 5 patients who had exhibited callus formation after 3 months which could no longer be examined during the follow-up after 6 months.

### Complications

Only one revision after 7 weeks due to infection was necessary. After surgical revision and the systemic administration of antibiotics, the soft tissue and the bone healed. In one case, one DLS 3.7 was removed after 4 months because of skin irritation. Bone healing itself was uneventful.

One year postoperatively, during implant removal in another hospital, one DLS 3.7 was found to be broken. In another case, the patient had no compliance and was already hiking after 4 weeks. The control X-ray which was realized after 1 year, showed the breakage of the three DLS 3.7 screws. In a third case, all three proximal screws were not inserted completely bicortically, but just into the cis-cortex, two screws were found to be broken during implant removal. In another case, the plate, which was fixed with a DLS 3.7 in the proximal part, was combined with a lag screw. After 3 months, the breakage of the lag screw was noted, probably as a result of the micro-motion enabled by the DLS 3.7. The overall complication rate (including all cases of screw breakage) in the distal tibia was 14.7%. The overall rate of screw breakage in the present study was 6.2%.

### Implant removals

A total of 12 implant removals in the distal tibia were uneventful and the screws could be extracted with the StarDrive Screwdriver without any problems.

## Discussion

In this study, we present the first clinical experiences with the use of the DLS 3.7 in distal tibia fractures and the technical innovations of this novel screw type in comparison to the well-known small-fragment Locking Head Screw (LHS 3.5). The aim of the study was to figure out the safety and effectiveness of the new DLS 3.7 in a limited clinical trial. The data were collected as part of a multi-center study. In this manuscript however, we only present the analysis of the results of the patients from our own center.

Concerning the problem of high stiffness in locking plate systems, different solutions have been presented in the past ten years [2,12-14,16-22]. However, the problem concerning the modulation of the fracture site movement in order to achieve the same range of motion despite the differences concerning the diameter of the treated bone and the length of the used screws remains unsolved. To date, the most extensive investigations about axial stiffness in locking plates have been carried out by Bottlang et al., Gardner et al., Duda et al., Cleas et al., Doebele et al., and Stoffel et al. [4,5,13,16-18,22]. In these studies, two questions of critical importance have been addressed:

– Does the high stiffness of the screw plate interface of locked plate systems suppress callus formation and fracture healing?

– Can different concepts increase fracture motion on the near cortex and improve fracture healing and are these concepts superior to the standard locking plating?

They found out that the interfragmentary motion of locked plates is asymmetrical: fragment motion in the cis-cortex region where the plate is located remains below the motion of 0.2-1 mm which is known to promote callus formation by continuous callus massage [9,16,23,24]. This is possibly one reason for the high rate of non-union (19%) and delayed union (37%) which has been found in studies describing distal femur fractures treated with locked plating osteosynthesis [20,25].

In order to solve the problem of an asymmetrical fracture motion of locked plating systems, several techniques have been introduced by different work group, like for example Far Cortical Locking, Dynamic Locking Screw, and near cortical slotted holes [16-18], with each technique having various advantages and disadvantages.

In a biomechnical study, Doebele et al. [16] have demonstrated in a biomechanical set-up that axial stiffness is reduced by using the DLS 3.7. In the same study, they have proven that a controlled symmetrical motion can be achieved, even if a bicortical fixation is required. This concept has promised to provide an interfragmentary motion of 0.45 mm, which is considered to be the ideal stimulus level for the promotion of secondary bone healing [3,6]. With the DLS 3.7, the established concept of the locking-plate osteosynthesis has remained untouched [16].

In the present study, all analyzed distal tibia fractures consolidated after at least 6 months. In spite of the limited number of 34 patients, these results are remarkable and better than those that can be found for the use of the LHS 3.5 with rates of delayed union or non-union up to 19% [19,25-27].

### DLS 3.7 related complications

Between 03/2009 and 10/2010, some problems with the insertion the diaphyseal DLS 3.7 into the trans-cortex, mainly in young patients with a thick cortex and a hard bone, were reported. The cutting performance of the DLS 3.7 was not as good as expected. However, by using additional flutes, the cutting performance was improved and even better results than with the LHS 3.5 were obtained.

The high expectations in the newly designed screw were subdued by the relatively high rate of secondary screw breakage of 11.8% (4 cases of 34). All these cases were intensively investigated by the surgeons and by the manufacturer (DePuy-Synthes, Zuchwil, Switzerland) and in none of them, a material-related breakage could be discovered. Including surgery reports, postoperative X-rays and patient history, it could be assumed that, in all cases, the surgical procedure or the compliance of the patient influenced the secondary implant failure. Some possible factors causing secondary implant failure are: full weight bearing already in the second week after treatment, insertion of the screw without a torque limiter and insufficient number of diaphyseal screws. This can be interpreted as an off-label-use and should be thoroughly considered during the assessment of the new implant, which, in our study, delivered excellent results.

Moreover, there already exists an international reference database concerning the rate of secondary implant failure with standard LHS 3.5. A similar database should be created for the DLS 3.7. An international index of secondary implant failure for screws could provide interesting data that could facilitate the impartial comparison of different implants.

For the use of the DLS 3.7 in distal tibia fractures, we issue the following recommendations:

– -A minimum of three DLS 3.7 is needed in the diaphyseal part,

– -The DLS 3.7 has to be inserted bi-cortically for proper function,

– -At least one DLS 3.7 has to be inserted close to the fracture in order to increase the bending stiffness of the plate system and to simultaneously decrease the axial stiffness of the osteosynthesis,

– -A torque limiter has to be used for the insertion of the DLS 3.7.

Based on these clinical experiences as well as on the results of the biomechanical and animal studies, the following recommendations can be made for the use of the DLS 3.7:

1. The DLS 3.7 can be indicated for metaphyseal fractures,

2. The principles of bridged plating regarding the length of the plate and the number of screws have to be complied to under all circumstances,

3. At least in the shaft area, the DLS 3.7 has to be used with one screw close to the fracture in order to increase the plate stiffness, and at the same time, in order to enable a greater reduction of the axial stiffness,

4. The DLS 3.7 should not be used with other screw types (LHS 3.5, conventional) in the same fragment.

Further clinical studies with higher numbers of cases are needed to show if this new concept is able to significantly reduce the rate of non-unions and delayed unions in distal femur and tibia fractures.

## Conclusions

This pilot observational cohort study could show that the DLS 3.7 in combination with locking compression plates provides a secure and easy application. According to the recent literature, adequate inter-fragmentary micro-motion is one evident goal to increase the reliability in fracture healing. The new DLS 3.7 with a maximum micro-motion of 0.2 mm combines the advantage of micro-motion with the well-known advantages of angular stable plate fixation.

### Consent

Written informed consent has been obtained from all patients for the publication of this case report and any accompanying images. A copy of the written consent is available for review for the editor-in-chief of this journal.

## Competing interests

The authors declare that they have no competing interests. None of the authors has any financial interests with regard to the companies whose products are described in this paper, i.e. DePuy-Synthes, Zuchwil, Switzerland.

## Authors’ contribution

All authors have contributed significantly to the different steps of the processing of the patient’s history as well as to the writing and to the editing of the manuscript. TF, SS, US and SD have conceived the idea for the study and have written the first draft. SS has provided research support and advice throughout the project. Furthermore, CEG has provided expertise in artwork. All authors have read and approved the final version of the manuscript.
